# Comparison between a new device for the semen quality analysis and the manual microscopic evaluation in a not specialistic clinical laboratory

**DOI:** 10.1515/almed-2024-0089

**Published:** 2024-08-26

**Authors:** Erika Jani, Margherita Bozzola, Elmar Marco Zagler, Vincenzo Roccaforte, Massimo Daves

**Affiliations:** Clinical Biochemistry Laboratory, Provincial Hospital of Bolzano (SABES-ASDAA), Bolzano, Italy; Clinical Pathology Laboratory, ASST Nord Milano, Sesta San Giovanni, Italy

**Keywords:** fertility, standardization, comparison, semen

## Abstract

**Objectives:**

Semen analysis investigates different parameters of human semen with a high relevance in fertility workup, confirmation of sterility by post vasectomy, in pathologies follow-up such as varicocele and in all cases where sperm preservation is required. Manually seminal fluid examination is characterized by poor reproducibility. Aim of this study was to evaluate the performance of an automatic device in semen analysis by comparing its results with those obtained with the manual microscopy.

**Materials:**

Fifty samples (age 18–59 years) were analyzed simultaneously by the manual and automated method. Manual analysis was performed by at least two experienced operators. Concentration and motility were determined by means of standard manual analysis and by the automated LensHooke™ analyzer following the last WHO guidelines.

**Results:**

We compared the concentration (million/mL) of spermatozoa obtained from manual and instrumental count and different classifications obtained: normal, oligospermic, cryptospermic and azoospermic samples. The Wilcoxon test does not show a statistically significant difference. The Bland-Altman plot showed a slightly higher value for the manual count. Second, we compared the morphology and the samples classification in morphological normal and abnormal. Third, spermatozoa motility obtained from the manual and instrumental count was compared with a different classification in normal total motility and asthenozoospermia. Statistical tests showed respectively for morphology and motility a moderate and a very good agreement.

**Conclusions:**

Our study demonstrates that the LensHooke™ shows an acceptable agreement with the manual microscopic seminal fluid evaluation. The use of this simple device could help to standardize reports in non specialistic laboratories.

## Introduction

Semen analysis is a diagnostic qualitative and quantitative exam which investigate different parameters of human semen with a high relevance in fertility workup, confirmation of sterility by post-vasectomy, in pathologies follow-up such as varicocele and in all cases where sperm preservation is required. Actually, manual sperm assessment (MSA) is the ‘gold standard’ to assess male fertility [1].

MSA consists of a macroscopic and a microscopic examination. Macroscopic assessment concerns the volume, appearance, the color of seminal fluid, and certain rheological characteristics such as viscosity and liquefaction. Microscopic evaluation concerns the concentration, motility and the morphology of spermatozoa. The MSA is crucial in the assessment of male fertility, but a full evaluation requires a complete medical history and other laboratory assessments (e.g., hormone tests) and imaging investigations [2].

Consequently, in clinical practice, semen quality analysis is the most frequently performed diagnostic evaluation and fundamental investigation in the management of the sub fertile couple and can guide the clinician to determine how to proceed with further investigation in an infertility workup 3], [4], [5.

The lower reference limits of sperm counts have been decreasing in recent decades, in parallel with longstanding debates on a global decline in human semen quality with equivocal findings and no emerging consensus so the World Health Organization (WHO) tried to provide reference values of semen parameters derived from several studies in different countries with a retrospectively and prospectively data. In this last edition of the WHO manual for semen analysis, the fifth centile values were newly proposed as the lower cutoff limits for normality. This value derived from the distribution of values from men who have contributed to a natural conception within 12 months of trying [1].

MSA, like most manually performed diagnostic examinations, is characterized by poor reproducibility due to subjective interpretation; this can affect the accuracy of the correct classification of the semen quality. Furthermore, MSA is a labor-intensive procedure and requires experienced and trained operators [3]. To overcome manually limitations, automatic device for the evaluation of semen quality would be welcome. Aim of this study was to evaluate the performance of an automatic device in semen analysis by comparing its results with those obtained with the manual microscopy method from different operators.

## Materials and methods

Fifty semen samples (age 18–59 years) were analyzed simultaneously by the manual and automated method over a period of consecutive 25 days (two samples per day). Manual semen analysis was performed by at least two experienced operators in our laboratory. Samples were collected after an abstinence period of 2–7 days and were delivered to the laboratory within 45 min following masturbation. Semen parameters, including concentration, motility and seminal pH, were determined by means of standard manual semen analysis and by the automated LensHooke™ X1 PRO semen quality analyzer following the WHO 6th Edition (2021) guidelines. Two different semen samples were evaluated every day for 25 consecutive working days. Sperm concentration (×10^6^/mL) was assessed using a Makler counting chamber after liquefaction of the semen sample in thermostat at 37 °C for 30–60 min. Sperm motility (%) was evaluated at room temperature by counting at least 100 spermatozoa using a light microscope at total magnification 250× (i.e., a combination of ×25 objective lens with a ×10 ocular). The spermatozoa were classified in four-category system, i.e., rapidly progressive, slowly progressive, non-progressive and immotile, as recommended by the sixth edition of WHO laboratory manual for the examination and processing of human semen. The morphology was evaluated using the microscope in phase contrast at total magnification 400× (i.e., a combination of ×40 objective lens with a ×10 ocular).

Automated semen analysis was performed using the LensHooke™ X1 PRO Semen Quality Analyzer (Bonraybio Co., Ltd, Taichung City, Taiwan) for sperm concentration, motility, and seminal pH in compliance with the World Health Organization (WHO) 6th Edition (WHO, 2021) and following the manufacture’s instruction.

The LensHooke^®^ X1 PRO Semen Quality Analyzer used with LensHooke^®^ Semen Test Cassette (Bonraybio Co., LTD,) is an optical device for human semen analysis which provides direct and calculated quantitative measurements for sperm concentration, sperm motility (progressive motility %, non-progressive motility %, immobility %), sperm morphology (normal form %) and pH value [6]. This instrument uses a high resolution and autofocus optical microscope in combination with an artificial intelligence-based interpretation system [3, 6]. Wilcoxon’s test was used to assess the statistical significance of differences between the results obtained by the two methods. A p<0.05 was set as statistically significant. To assess the degree of agreement between the results obtained with the two different methods in sperm concentration and total motility, we used the Bland-Altman plot and the Passing and Bablok regression and for qualitative agreement for categorical values the weighted kappa coefficient. Statistical analysis was performed with MedCalc17.4.4^©^ statistical software (MedCalc Software, Ostend, Belgium).

The study was conducted according to the latest version of the Declaration of Helsinki given by the World Medical Association. Formal approval of the protocol by the local Ethics Committee was considered unnecessary because the assessment of semen analysis was part of the routine examinations prescribed to patients and the study did not interfere with the usual clinical routine and all results have been anonymized before evaluation.

## Results

**Figure 1:**
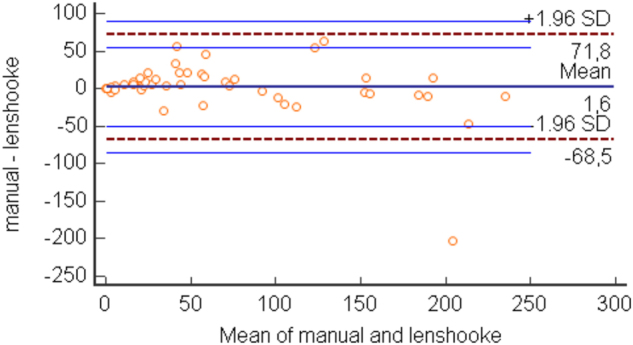
Bland-Altman plot comparing the sperm concentration measured with manual and instrument count. Mean difference: 1.6 million/mL.

First, we compared the concentration (million/mL) of spermatozoa obtained from manual and instrumental counts. For both counts the D’Agostino-Pearson Test for Normal distribution reject Normality. The median value obtained with the manual count was 50.5 million/mL, 95 % Confidence Interval (CI) for the median 27.60–75 (highest value 230 million, lowest value 0), Interquartile Range (IQR) 20–95 million/mL. With the LensHooke instrument we observed a median value of 35 million/mL, 95 % CI 19.9–66.27 (highest value 306 million, lowest value 0.3), IQR 13.4–97.3. The Passing and Bablok regression was Y=−4.03 + 1.01X, (intercept −4.03; 95 % CI: −7.31 to −0.47; slope 1.01, 95 % CI: 0.89–1.09) were Y=Lenshooke instrument and X=manual count. Spearman's rank correlation coefficient 0.944. The Wilcoxon test does not show a statistically significant difference. The Bland-Altman plot comparing the manual and instrumental count showed a little positive bias, indicating a slightly higher value for the manual count (Figure 1).

We also wanted to compare the different classifications obtained with the two counts in normal (spermatozoa concentration>16 million/mL), oligospermic (<15 million/mL), cryptospermic (spermatozoa concentration<1 million/mL) and azoospermic (without spermatozoa) samples. Results are reported in Table 1; the weighted kappa was 0.761 (95 % CI −0.585 to 1.000) which represents a good agreement. Of note that the 5th edition implies the loss of this nomenclature with the introduction of the term “Decision limit” to describe deviation from semen reference values. Second, we compared the morphology and the different samples classification in morphological normal and abnormal (teratospermia: abnormal morphological sperm>96 %) samples. Table 2 shows the different classification according to morphology with a weighted kappa of 0.52 (95 % CI −1.000 to 1.000) which represents a moderate agreement.

**Table 1:** Different sample classification in according to sperm concentration; weighted kappa: 0.761.

	Manual count				
Lenshooke	Azo	Cripto	Oligo	Normo	
Azo	0	0	0	0	0
Cripto	0	1	0	0	1 (2 %)
Oligo	0	0	0	38	38 (76 %)
Normo	1	1	9	0	11 (22 %)
	1 (2 %)	2 (4 %)	9 (18 %)	38 (76 %)	50

Azo, azoospermia; Cripto, criptozoospermia; Oligo, oligozoospermia; Normo, normozoospermia.

**Table 2:** Different sample classification in according to sperm morphology; weighted kappa: 0.520.

	Manual count		
Lenshooke	Normal	Terato	
Normal	28	1	29 (58 %)
Terato	10	11	21 (42 %)
	38 (76 %)	12 (4 %)	50

Terato, teratozospermia.

Third, we compared the spermatozoa motility obtained from the manual and instrumental count and the different classification in normal total motility and asthenozoospermia. A sample with normal motility according to the latest WHO indications must have at least 42 % motile spermatozoa. For the total motility (fast progressively motile, slow progressively motile, non-progressively motile expressed as a percentage of the total number of spermatozoa) the median value obtained with the manual count was 55.5 %, 95 % CI for the median 48.21−68.59 (highest value 90 %, lowest value 0 %), IQR 37–77 %. With the LensHooke instrument we observed a median value of 60.5 %, 95 % CI 41.21–72 (highest value 99 %, lowest value 3 %), IQR 30–76. The Wilcoxon test does not show a statistically significant difference. The Passing and Bablok regression was Y=2.64 + 1.04X, were Y=Lenshook instrument and X=manual count, intercept 2.64 (95 % CI: −13.26 to 11.24), slope 1.04 (95 % CI: 0.84–1.35). Spearman's rank correlation coefficient 0.688. Table 3 shows the different classification according to total motility with a weighted kappa of 0.839 (95 % CI −1.000 to 1.000) which represents a very good agreement.

**Table 3:** Different sample classification in according to sperm total motility; weighted kappa: 0.839.

	Manual count		
Lenshooke	Astheno	Normal	
Astheno	14	0	14 (28 %)
Normal	4	32	36 (42 %)
	18 (36 %)	32 (64 %)	50

Astheno, asthenozoospermia.

## Discussion

The WHO laboratory manual for the examination and processing of human semen is considered to be the main reference for the laboratories engaged in semen fluid analyses. However, the interpretation and application of previous WHO ‘normal’ or ‘reference’ values for semen parameters used have limitations since the data were derived from imprecisely defined reference populations and obtained from different laboratories with different analytical methodologies [7].

It seems very challenging to identify individual semen parameters predicting the likelihood of pregnancy also in case of assisted reproduction. This can be explained by a lack of standardization of semen analysis derived by different methodologies and different experiences [8, 9].

The major shortcomings of standardizing of MSA are due to subjectivity of this test and its dependence on skills of the operator. The latter is of considerable importance in a general clinical laboratory, where the performance of seminal fluid examination represents a modest part of work activities in contrast to laboratories specializing in infertility diagnostics. The introduction of an instrument that can ensure greater standardization in the performance of seminal fluid examination is certainly an improvement in laboratory practice, especially in non-specialist laboratories. In our study we evaluated the results obtained with the LensHooke X1 analyzer in the sperm quality evaluation in comparison with the traditional manual microscopy evaluation. The most important aspect we wanted to test with the instrument was its ability to classify patients according to spermatozoa concentration, morphology and motility in accordance with the classification obtained with the manual microscope method. Results of our work agree with those published by other authors which concluded that spermatozoa concentration and motility data derived from the LensHooke analyzer are comparable with the manual microscopic evaluation [5]. For the spermatozoa concentration, the Bland-Altman plot comparing the manual count with the instrumental count showed a little positive bias, indicating a slightly higher value for the manual count as it is shown also by the Passing-Bablok regression that demonstrate furthermore the absence of a proportional bias (Y=−4.03 + 1.01X; intercept −4.03; 95 % CI: −7.31 to −0.47; slope 1.01, 95 % CI: 0.89–1.09 were Y=Lenshooke instrument and X=manual count). The comparison of spermatozoa motility obtained from the manual and instrumental count and the different classification from normal total motility to asthenozoospermia appears more than acceptable as underlined by the value of weighted kappa of 0.839. Furthermore, we evaluated the grade of concordance in the morphological assessment between these two different methods considering that distinctions between morphologically normal and abnormal spermatozoa therefore will continue to be important factors that must be considered in clinical practice on human sperm function [8, 9]. We obtained a weighted kappa of 0.52 which represents a moderate agreement. This is not surprising considering the big methodological difference between microscopic and the automatic instrument evaluation: the first is based on observation with the operator’s eye so results interpretation is experience-dependent; the second is based on a precise algorithm which cross-analyzes spermatozoa’s head and tail dimensional measurements and classified them. In any case, the degree of concordance that we obtained appears acceptable for a first assessment of semen quality performed in a laboratory not specialized in assessing infertile couples. In addition, the instrument’s small size and ease of use make it suitable for the needs of non-specialist laboratories. Besides a better sensitivity, another major advantage of this technique is the easiness of evaluation and interpretation compared to optical microscopic evaluation.

Our study is an explorative study with limitations that should be highlighted; first of all, the small number of samples used for the comparison; certainly, further studies with a higher samples number should be necessary to clarify the correlation between manual and automated method. Another important limitation is that we did not use the recommended staining for the morphology assessment as recommended by the last WHO guidelines but the phase-contrast microscopic observation for the evaluation of spermatozoa morphology. This procedure is not in line with the WHO’s latest recommendations for the assessment of spermatozoa morphology, which requires the preparation of a smear of ejaculate on a slide, fixation and staining with the Papanicolau staining [1, 5]. The eventual use of other staining should be validated in comparison with the reference Papanicolau staining [1, 5]. In our laboratory we have carried out in recent years a comparison of the morphology obtained in phase contrast with that obtained after staining the slide so we can still use the microscope in phase contrast as was permitted by old recommendations. Another limitation was that we do not use post-vasectomy samples to evaluate if the LensHooke™ X1 PRO is able to detect spermatozoa or not in these cases.

In conclusion the LensHooke™ X1 PRO automated semen analyzer shows an acceptable agreement with the manual microscopic evaluation of seminal fluid coupled to the easiness of technique implementation, these attractive features suggest the opportunity for a wider adoption of this instrument technique in the clinical practice. The use of this simple device could help to standardize reports and clinical information in first level laboratories semen analysis.
